# Improved Production of Recombinant Myrosinase in *Pichia pastoris*

**DOI:** 10.3390/ijms222111889

**Published:** 2021-11-02

**Authors:** Zuzana Rosenbergová, Zuzana Hegyi, Miroslav Ferko, Natália Andelová, Martin Rebroš

**Affiliations:** 1 Institute of Biotechnology, Faculty of Chemical and Food Technology, Slovak University of Technology, Radlinského 9, 812 37 Bratislava, Slovakia; zuzana.rosenbergova@stuba.sk (Z.R.); zuzana.hegyi@stuba.sk (Z.H.); 2 Centre of Experimental Medicine, Institute for Heart Research, Slovak Academy of Sciences, Dúbravská cesta 9, 841 04 Bratislava, Slovakia; usrdmife@savba.sk (M.F.); usrdnata@savba.sk (N.A.)

**Keywords:** signal sequence, *Pichia pastoris*, myrosinase, *Arabidopsis thaliana*, plant enzymes

## Abstract

The effect of the deletion of a 57 bp native signal sequence, which transports the nascent protein through the endoplasmic reticulum membrane in plants, on improved *At*TGG1 plant myrosinase production in *Pichia pastoris* was studied. Myrosinase was extracellularly produced in a 3-liter laboratory fermenter using α-mating factor as the secretion signal. After the deletion of the native signal sequence, both the specific productivity (164.8 U/L/h) and volumetric activity (27 U/mL) increased more than 40-fold compared to the expression of myrosinase containing its native signal sequence in combination with α-mating factor. The deletion of the native signal sequence resulted in slight changes in myrosinase properties: the optimum pH shifted from 6.5 to 7.0 and the maximal activating concentration of ascorbic acid increased from 1 mM to 1.5 mM. Kinetic parameters toward sinigrin were determined: 0.249 mM (K_m_) and 435.7 U/mg (V_max_). These results could be applied to the expression of other plant enzymes.

## 1. Introduction

Over the last two decades, proteins have become very important substances in many industrial, medical and research fields. The protein products range from industrial and diagnostic enzymes to protein-based drugs and vaccines. One of the main obstacles is the production of the desired protein products in sufficient amounts [1]. Recombinant production of proteins is often used to produce large amounts of proteins needed for industrial and pharmaceutical applications. One of the most widely used hosts for protein expression is the Gram-negative bacterium *Escherichia coli*, due to its rapid multiplication, inexpensive nutritional requirements, fast and easy transformation, and high-level expression of the recombinant protein. However, the frequent misfolding and aggregation of recombinant proteins is one of the main disadvantages of this expression host [2]. Moreover, this expression system cannot be used for the production of proteins requiring posttranslational modifications (e.g., glycosylation), which are crucial for the correct folding and activity of eukaryotic proteins.

Glycosylation is one of the key properties of recombinant proteins with pharmaceutical or medical application—glycoprotein drugs have been used against autoimmune disorders, cancers, and invasive diseases caused by pathological microorganisms. It has been reported that N-glycosylation and N-glycan structures can affect biophysical and pharmacokinetic properties of therapeutic proteins [3]. Moreover, proteins with incorrect glycosylation can cause immunological responses in a target organism. The majority of pharmaceutical proteins are therefore produced in Chinese hamster ovary (CHO) cells, which perform human-like glycosylation. However, protein production in CHO is very expensive and requires complex growth media, which are often contaminated with animal viruses during the production of recombinant proteins [4].

One of the most popular alternative hosts for the production of glycosylated proteins is the methylotrophic yeast *Pichia pastoris*. It offers low-cost cultivation in minimal media, which can be easily upscaled to a large scale. Proteins are correctly folded and glycosylated in a pattern similar to mammalian cells. *P. pastoris* is able to perform O- and N-glycosylation [5], the latter having been analyzed for many years and, in the end, its humanization was achieved through glycoengineering [6]. Moreover, *P. pastoris* is a suitable system for the production of secreted proteins, because it not only processes signal peptides correctly but also secretes a limited amount of endogenous proteins to the culture supernatant [1]. To this day, many pharmaceutically relevant protein products have been produced in *P. pastoris*, e.g., proinsulin [7], human epidermal growth factor [8], apidaecin [9] and other antimicrobial peptides, and subunit vaccines [1].

One of the proteins with potential pharmaceutical applications is the plant defense enzyme myrosinase (EC 3.2.1.147). At neutral pH, myrosinase hydrolyses the plant secondary metabolites glucosinolates to isothiocyanates [10]. This group of compounds is of particular interest because isothiocyanates have been shown to have anticarcinogenic and antitumorigenic effects. Moreover, their toxicity to regular cells is low which makes isothiocyanates promising agents in cancer prevention and treatment [11]. Myrosinases have been identified in many plants, fungi, and several gut microbes. Plant myrosinases are especially hard to purify because they tend to associate with lectin-like proteins. Moreover, more than one myrosinase isoenzyme is usually present in a single plant tissue [12]. Recombinant production of myrosinases in *P. pastoris*, on the other hand, represents a simple and inexpensive way of producing a single myrosinase isoenzyme which can be easily purified from the culture supernatant.

Recently, the recombinant production of TGG1 myrosinase from *Arabidopsis thaliana* in *Pichia pastoris* has been upscaled and optimized [13]. However, the genetic aspect of recombinant myrosinase production has not been studied. It has been previously reported, that myrosinases contain N-terminal signal sequences which are needed for the nascent polypeptide chain to traverse the endoplasmic reticulum membrane. It was suggested that myrosinase is anchored to membranes by an uncleaved signal peptide, which was supported by the fact that a fraction of myrosinase was membrane associated [14]. Therefore, the improper cleavage of the signal peptide by *P. pastoris* could result in the attachment of the recombinant myrosinase to host cell membranes, which would lower the yield of secreted enzymes.

In this study, the effect of the deletion of a native signal sequence from *At*TGG1 myrosinase gene on its expression in *Pichia pastoris* was studied. Myrosinase was produced in a high-cell density fermentation using an optimized protocol [13] and purified using ion-exchange chromatography. The purity of the enzyme was confirmed by liquid chromatography-tandem mass spectrometry analysis.

## 2. Results and Discussion

### 2.1. The Overexpression of Myrosinase without Native Signal Peptide

The *At*TGG1 myrosinase gene was expressed in *P. pastoris* in our previous study [13]. Although the high-cell density fermentation was optimized and the specific productivity increased 4-fold, the amount of myrosinase produced was still low. The sequence of the gene, which was cloned into the pPICZαA vector, was analyzed with the SignalP 5.0 prediction program [15]. The analysis confirmed that the expressed gene contained an N-terminal native signal sequence encoding a 19-amino acid signal peptide. In 1993, Thangstad et al. [14] described the nucleotide sequences of the *Myr1* and *Myr2* genes from *Brassica napus*, which contained signal sequences encoding 19 and 20 amino acid long signal peptides, respectively. These signal peptides, which possess a positively charged N-terminus followed by a hydrophobic h-region, are needed for the nascent polypeptide chain to traverse the endoplasmic reticulum membrane. They are later cleaved from the nascent chain, releasing the mature protein [14,16]. Previously, Andersson et al. [17] reported intracellular expression of three myrosinases (TGG1, TGG4, and TGG5) from *Arabidopsis thaliana*, which were cloned into a pPIC3.5K vector with their respective native signal sequences to ensure the produced enzymes entered the secretory pathway of *P. pastoris* [17]. Wang et al. [18] also reported intracellular expression of myrosinase from *Carica papaya* with its 24-amino acid signal peptide, which was successfully cleaved during the production of myrosinase in *P. pastoris* [18]. These results suggest that *P. pastoris* is able to process the native signal peptide correctly. However, if the signal peptide processing is incomplete, myrosinase could be attached to host-cell membranes through the uncleaved signal peptide, which would lower the yield of the secreted myrosinase.

**Figure 1:**
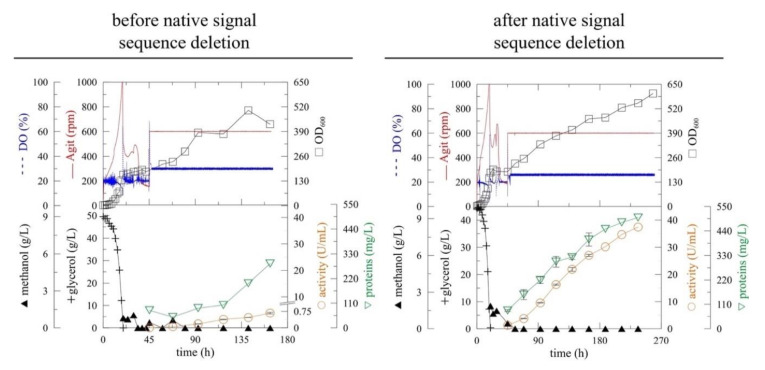
The improvement of (α-MF)-driven secretion of myrosinase in *Pichia pastoris* before (*myr*) [13] and after (*myr*-Δ19) the deletion of the native N-terminal signal sequence. The expression was performed in *Pichia pastoris* KM71H in a 3-L laboratory fermenter at 20 °C, pH 6 [DO (Dissolved oxygen), Agit (agitation), OD_600_ (optical density measured at 600 nm)].

Therefore, the 57 base pair long signal sequence was deleted from the *At*TGG1 gene using PCR. The PCR product was then cloned into the pPICZαA vector downstream of the α-MF secretion signal and transformed into *P. pastoris* KM71H (Mut^S^) strain. The best myrosinase expressing clone (selected out of 88 clones) was cultivated in a 3-liter laboratory fermenter (Figure 1), according to our previous study. After glycerol depletion (approx. 20 h), the strain was adapted to methanol by two methanol additions to 3 g/L of final concentration. The continual methanol feeding, which was based on the actual concentration of dissolved oxygen (DO), was started at 45 h of fermentation. The expression was performed at 20 °C and pH 6 to reduce cell stress and product proteolysis [13].

**Figure 2:**
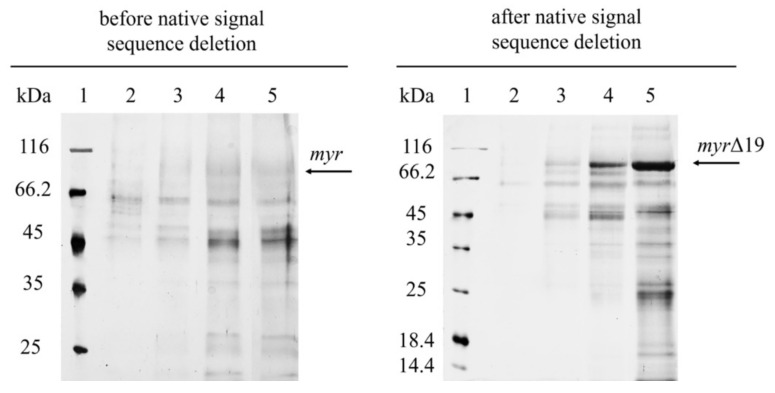
SDS-PAGE analysis of cell-free supernatants from the fermentation of *P. pastoris* expressing *myr* and *myr-*Δ19 (after native signal sequence deletion): lane 1—molecular weight marker; lane 2–5—cell-free supernatant taken after 45 h (2); 69 h (3); 140 h (4); 164 h (5) of fermentation.

The deletion of the native signal sequence markedly improved recombinant myrosinase (*myr*-Δ19) production (Figure 1)—both the volumetric activity and specific productivity increased more than 40-fold after 164 h of fermentation (Table 1). The volumetric activity of myrosinase increased linearly until the end of the fermentation, however, the maximum of specific productivity was achieved at 164 h of fermentation. The improvement in myrosinase yield was also shown by SDS-PAGE electrophoresis (Figure 2), where an 85 kDa band was visible for *myr*-Δ19, while only an indistinct band was detected during the production of myrosinase with its signal peptide (*myr*).

The improvement in recombinant protein production after the deletion of signal sequences has been reported before. Yang et al. [19] studied the influence of native signal sequences in the transgene on (α-MF)-driven protein secretion in *P. pastoris*. The deletion of both the N- and C-terminal native signal peptide sequences of alkaline phytase enhanced its (α-MF)-driven secretion in *P. pastoris* by 4-fold [19]. It has been previously reported that the selection of signal peptides is crucial for successful protein expression in *P. pastoris* and needs to be optimized for each protein individually [20,21].

### 2.2. The Purification of Myrosinase

**Figure 3:**
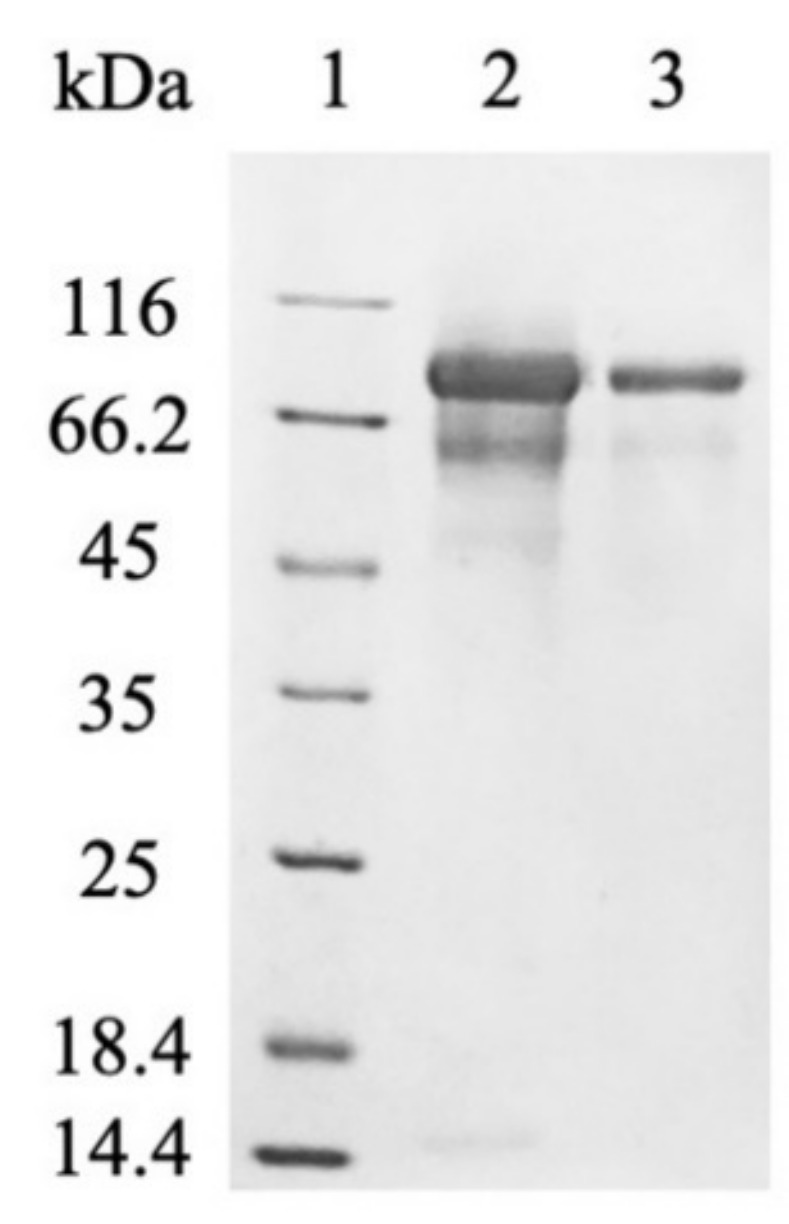
SDS-PAGE analysis of *myr-*Δ19 after purification: lane 1—molecular weight marker; lane 2—ion-exchange chromatography with DEAE-Sepharose, lane 3—subsequent desalting on a 30 kDa cut-off membrane.

*P. pastoris* is one of the most popular hosts for the extracellular production of recombinant proteins due to the low secretion of endogenous proteins, which greatly simplifies the purification of the desired protein [5]. However, the cell-free supernatant after fermentation was contaminated with many proteins (Figure 2). Most of them were successfully removed by anion-exchange chromatography [modified from Härtel and Brandt [22]] and subsequent desalting on a 30 kDa cut-off membrane (Figure 3). The purification of *myr* was also attempted, however, only partially purified myrosinase was obtained. The purity did not improve with subsequent purification, which also resulted in decreased myrosinase activity (data not shown). This problem was most likely caused by a large amount of contaminating proteins being attached to the column that could not have been separated from *myr*. After the deletion of the native signal sequence, the amount of myrosinase in the supernatant increased substantially (Figure 2) and the majority of contaminating proteins did not attach to the column. The deletion of the signal sequence, therefore, facilitated myrosinase production as well as its purification.

**Figure 4:**
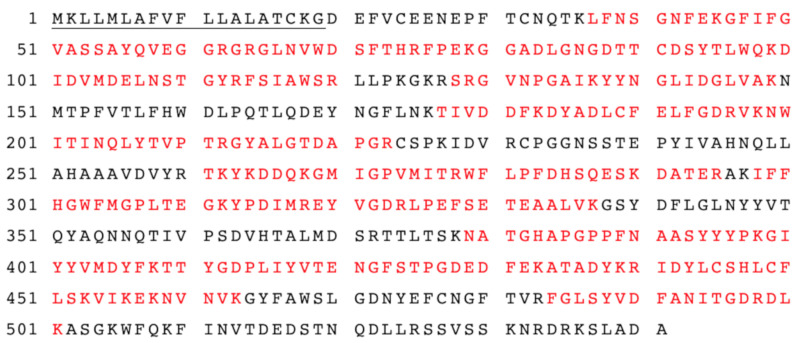
The amino acid sequences of *At*TGG1 myrosinase identified by LC-MS-MS analysis. The amino acid sequence coverage of myrosinase (*myr-*Δ19) with identified peptides was 61%. Matched peptides shown in red. The underlined amino acids represent the native signal peptide.

However, one protein with a molecular mass of approximately 60 kDa remained in the purified *myr*-Δ19 fraction and could not be removed by further purification with ion-exchange and size-exclusion chromatography (data not shown). In order to determine the identity of this protein, the purified fraction was digested with trypsin and analyzed with liquid chromatography-tandem mass spectrometry analysis (LC-MS/MS) according to Andelová et al. [23]. Interestingly, myrosinase was identified with 59 peptide fragments as a sole protein in the purified fraction. The sequence coverage of myrosinase is shown in Figure 4. These results suggested that both bands visible on the SDS-PAGE gel (Figure 3) represented a myrosinase, but with different degrees of glycosylation.

The majority of the produced myrosinase appeared as an 85 kDa band on SDS-PAGE gels, which is almost 10 kDa more than previously published results for the subunit mass of intracellularly produced *At*TGG1 myrosinase in *P. pastoris* GS115 [17] and *At*TGG1 myrosinase produced in plants [24]. The difference in size was probably the result of more extensive glycosylation either as a result of the extracellular expression or a different production strain. Previously, Härtel and Brandt [21] reported the extracellular production of MYR1 myrosinase from *Brassica napus* in *P. pastoris* GS115 (Mut^+^). The size of the produced myrosinase corresponded well with the size of its glycosylated form, although a micro-heterogeneity of the pI was observed [22]. However, a difference in glycosylation patterns between different *P. pastoris* strains has been previously reported [25], suggesting that the production strain itself could have affected myrosinase glycosylation.

Overall, 124 mg of pure myrosinase were obtained from 1 L of culture supernatant, which is 62-times higher than the amount of pure *At*TGG1 myrosinase obtained by Andersson et al. [17] and 1.7-times higher than the amount of native *At*TGG1 myrosinase obtained from 1 kg of leaves from mutant *Arabidopsis* lines [24].

### 2.3. The Hyper-Glycosylation of Recombinant Myrosinase

To this day, myrosinase from *Sinapis alba* is the only myrosinase to be studied by X-ray crystallography [26]. Later, in silico models of myrosinases from *Brassica oleracea* [27] and *Brassica juncea* [28] were constructed. Based on these studies, myrosinases are stabilized by three disulfide bonds, a large number of salt bridges, and carbohydrate residues that are distributed over the entire protein surface [12]. It was hypothesized that such extensive glycosylation was needed to maintain solubility and molecular stability of myrosinase enzymes since many of them were localized in a dehydrated environment of the seeds [26]. It is estimated that N-glycosylation also protects myrosinases from reactive hydrolysis products and facilitates complex formation with myrosinase binding proteins found in plants [12]. The number of glycosylation sites, represented by the conserved sequence Asn-Xxx-Thr/Ser, varies across the myrosinase family—seven N-glycosylation sites were predicted for myrosinase from *Brassica juncea*, ten for *Sinapis alba* [28]. It was previously reported that native *At*TGG1 myrosinase contains nine glycosylation sites and is extensively glycosylated [29].

**Figure 5:**
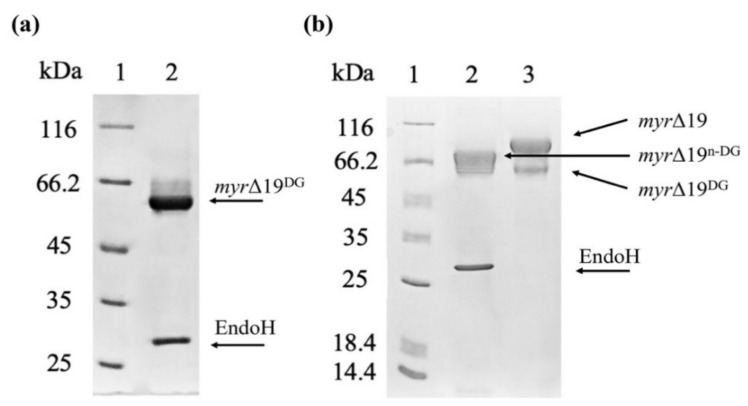
The deglycosylation of a purified myrosinase (*myr*-Δ19) with EndoH. (**a**) SDS-PAGE analysis of myrosinase deglycosylation under denaturing conditions: lane 1—molecular weight marker; lane 2—deglycosylated myrosinase (*myr*-Δ19^DG^). (**b**) SDS-PAGE analysis of myrosinase deglycosylation under non-denaturing conditions: lane 1—molecular weight marker; lane 2—deglycosylated myrosinase (*myr*-Δ19^n-DG^), lane 3—purified *myr*-Δ19 myrosinase.

In order to determine the effect of this hyper-glycosylation of *myr*-Δ19 on myrosinase activity, *myr*-Δ19 was deglycosylated with Endo-β-N-acetylglucosaminidase H (EndoH) (New England Biolabs, Ipswich, MA, USA), which cleaves the GlcNAcβ-1,4-GlcNAc linkage in the core of high-mannose oligosaccharides. Endo H has been widely used to determine the N-linked glycosylation on proteins using gel-shift assay [30]. Heat-denatured myrosinase (*myr*-Δ19) was completely deglycosylated with EndoH (*myr*-Δ19^DG^) as its subunit mass was reduced to approximately 60 kDa corresponding to the mass of the naked polypeptide chain (59.1 kDa, as calculated by EXPASY, https://web.expasy.org/protparam/, accessed on 15 September 2021). As a result, only one myrosinase band was visible on the SDS-PAGE gel (Figure 5a). Under non-denaturing conditions, myrosinase (*myr*-Δ19^n-DG^) subunit mass decreased only to 75 kDa (Figure 5b), probably due to steric hindrances. This size corresponds to the mass of native myrosinase isolated from *A. thaliana* [29], confirming that myrosinase is hyper-glycosylated by *P. pastoris* KM71H strain.

An in-depth analysis of native *At*TGG1 glycosylation patterns was performed by Liebminger et al. [29], who reported that all nine glycosylation sites in *At*TGG1 myrosinase were glycosylated with Man_5_GlcNAc_2_ as the major glycoform [29]. While *P. pastoris* is able to carry out N-glycosylation of the amide nitrogen of asparagine residues in the conserved Asn-Xxx-Thr/Ser sequence, the most common oligosaccharide being assembled on *P. pastoris* produced proteins is Man_14_GlcNAc_2_, resulting in hyper-glycosylation of the produced enzyme. However, the hyper-glycosylation is far less extensive compared to more than 50 mannose residues added to proteins by *S. cerevisiae* [30].

It has been previously reported, that N-glycosylation can affect enzyme properties and stability. Pérez de los Santos et al. [31] reported slight changes in INVA and INVB invertases properties after deglycosylation with EndoH. While the temperature optimum of INVB did not change, the temperature optimum of INVA decreased by 5 °C. Moreover, the kinetic parameters and operational stability of both enzymes changed after deglycosylation, suggesting that carbohydrates protect enzymes from heat denaturation and premature aggregation [31]. The research on the effects of N-glycosylation on recombinant endoglucanase IIa from *Pencillium verruculosum* has shown that the enzyme properties (pH and temperature optima) were very similar for all mutant enzymes (each carrying an Asn to Ala substitution in the predicted N-glycosylation site). However, differences were observed in terms of specific activity and kinetic parameters of the mutant enzyme [32].

Recently, the effect of different N-glycosylation patterns on the properties of feruloyl esterase 1a from *Myceliophtora thermophila* was determined [33]. The enzyme was produced in its native producer as well as in *P. pastoris* GS115. The recombinant enzyme exhibited a slightly higher mass on SDS-PAGE gel (33 kDa vs. 31 kDa for a subunit). While the overall catalytic efficiency has not been affected by different glycosylation, the temperature and pH stability of the natively glycosylated enzyme was higher than its recombinant counterpart. The difference in feruloyl esterase properties was apparent despite the enzyme containing only two glycosylation sites [33]. The effect of hyper-glycosylation on enzyme properties can be even more significant with an increased number of glycosylation sites (nine glycosylation sites in *At*TGG1 myrosinase). However, the effect of hyper-glycosylation on the activity of recombinant myrosinase was negligible—the specific activity of *myr*-Δ19 increased by only 13% after EndoH treatment under non-denaturing conditions (*myr*-Δ19^n-DG^). The slight decrease in myrosinase activity that is caused by hyper-glycosylation can be compensated with high amounts of the enzyme that are produced in *P. pastoris* compared to very difficult and time-consuming production and isolation of myrosinases from plants.

### 2.4. The Catalytic Properties of Produced Myrosinase

**Figure 6:**
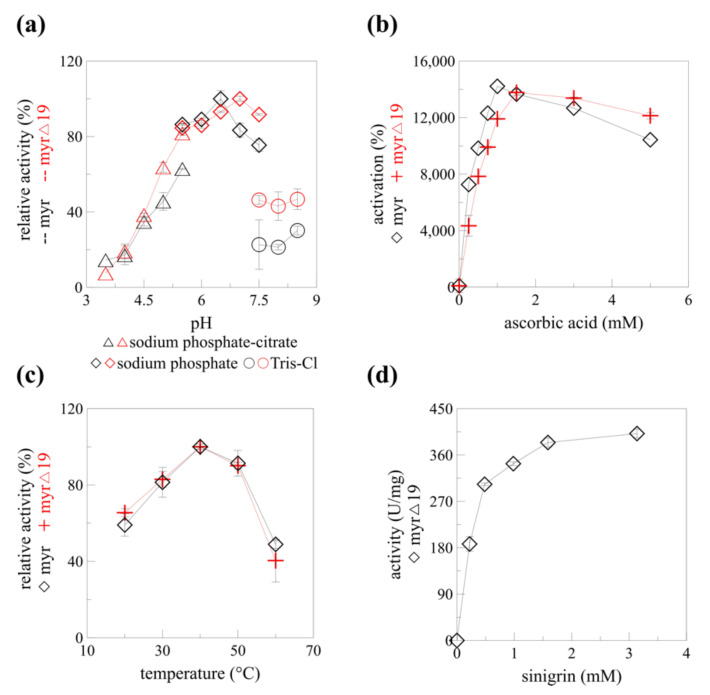
The enzyme characteristics of myrosinase before (*myr*) and after (*myr*-Δ19) native signal sequence deletion. (**a**) pH profile, (**b**) ascorbic acid-dependent activation, (**c**) temperature profile of *myr* [13] and *myr*-Δ19. (**d**) Kinetics of *myr*-Δ19 with different concentrations of sinigrin and 1.5 mM of ascorbic acid as activator, measured at 40 °C, pH 7.

The catalytic properties of recombinant myrosinase changed slightly after the native signal sequence deletion (*myr*-Δ19). The optimum pH shifted from 6.5 to 7.0 in sodium phosphate buffer (Figure 6a) which is beneficial for myrosinase pharmaceutical application. Isothiocyanates, compounds with a number of medical applications including cancer treatment and prevention, are produced from glucosinolates at pH 7 [10]. The maximal activating concentration of ascorbic acid increased from 1 mM to 1.5 mM (Figure 6b). Interestingly, the temperature profile did not change after the deletion of the signal sequence (Figure 6c). A slight change in pH optimum (0.5 units) to the neutral region was reported for endoglucanase IIa for two out of three mutant enzymes with altered N-glycosylation. Similarly to his paper, the temperature optimum was not affected [32]. Previously, recombinant α-L-rhamnosidase produced in *P. pastoris* KM71H was shown to be hyper-glycosylated. While the characteristics of the native and recombinant α-L-rhamnosidase and their EndoH deglycosylated counterparts were slightly different (pH optimum, temperature stability), the bioconversion of rutin to isoquerticin was not affected [34]. It is clear, that a detailed structural analysis would be required to determine any changes in the myrosinase structure caused by the signal peptide absence and de/glycosylation, and their effect on the biocatalytic properties of different forms of recombinant myrosinase: *myr*, *myr*-Δ19, and *myr*-Δ19^n-DG^.

The mechanism of myrosinase hydrolytic activity was solved more than 20 years ago [35]. Unlike the rest of glycosidases, myrosinases possess only one glutamate residue in their active site (Glu401 in *At*TGG1), the other glutamate was replaced by glutamine (Gln186 in *At*TGG1) during evolution to prevent unfavorable electrostatic interactions of glutamate with the sulfate group of glucosinolates [12]. The catalytic activity of the missing glutamate was substituted by ascorbic acid. This deglycosylation step of the myrosinase catalyzed hydrolysis was found to be rate-limiting and is activated by ascorbate [35]. A more than 140-fold increase in myrosinase activity was observed with 1 mM ascorbic acid [13] for *myr* and 1.5 mM ascorbic acid for *myr*-Δ19 (Figure 6b). A similar increase in V_max_ was observed for myrosinase from *Raphanus sativus* seedlings [36]. Since the specific activity of myrosinase without ascorbic acid is very poor, it was decided to determine the kinetic parameters of *myr*-Δ19 only in the presence of ascorbic acid.

Substrate saturation was reached at a concentration of 1.5 mM sinigrin. According to the Hanes plot, the K_m_ value was 0.249 mM, which is more than two times higher than the value determined for recombinant *At*TGG1 myrosinase produced in plants; the maximum velocity (V_max_) towards sinigrin was 435.7 U/mg, more than 40-times higher than previously reported results [24]. Our results varied greatly from the ones obtained by Andersson et al. [17]. Substrate saturation was reached already at 42 μM of sinigrin for *At*TGG1 myrosinase and the maximum velocity reached only 2.2 U/mg according to Hanes plot. The kinetic measurements were, however, performed without ascorbic acid [17]. Taking into account the maximal activation reported by Andersson et al. [17], the maximal specific activity (V_max_) would be still four times lower than the one reported in this paper. To our knowledge, the specific activity of 435.7 U/mg reported in this paper is the highest specific activity reported for any of the myrosinase enzymes while using sinigrin as a substrate.

## 3. Materials and Methods

### 3.1. Signal Peptide Deletion and Recombinant Strain Preparation

A partial DNA sequence of *At*TGG1 was amplified from the plasmid pPICZαA-*myr* used in our previous study [13]. A sense primer (5′-ATGTAGAATTCGATGAATTTGTTTGTGAAGAAAA-3′) containing the *Eco*RI site and antisense primer (5′-ATGTGGTACCTAAGGCGTATCCA-3′) containing the *Kpn*I site were designed to amplify a DNA sequence ranging from nucleotides 58 to 654 of the original *At*TGG1 gene. The PCR was performed using Q5^®^ High-Fidelity DNA Polymerase (New England Biolabs, Ipswich, MA, USA) and was carried out for 30 cycles with conditions as follows: 98 °C for 7 s, 56 °C for 20 s and 72 °C for 35 s. The 603 bp-long PCR product was double-digested with *Eco*RI/*Kpn*I and ligated into pPICZαA-*myr*, also digested with *Eco*RI/*Kpn*I. The resulting recombinant plasmid (pPICZαA-*myr*Δ19) was verified by DNA sequencing. The plasmid pPICZαA-*myr*Δ19 was transformed into competent *P. pastoris* KM71H cells according to Lin-Cereghino et al. [37]. Detailed information about the recombinant strain preparation and the selection of the best expressing clone can be found in Rosenbergová et al. [13].

### 3.2. Overexpression of Recombinant Myrosinase (myr-Δ19)

The high-cell density production of myrosinase was performed as previously described [13,38]. The fermentation was performed in a 3 L fermenter (New Brunswick^TM^ BioFlo^®^ 115, Eppendorf, Hamburg, Germany) with 1.5 L BSM medium supplemented with 6.525 mL of PTM_1_ trace salts solution (the media compositions are available in Supplementary Materials). The BSM medium was inoculated (5% (*v*/*v*)) with an overnight culture of *P. pastoris* KM71H grown in BMGY medium at 30 °C, 200 rpm. The fermentation conditions were as follows: 20% DO (dissolved oxygen) saturation was maintained with agitation cascade (50–1000 rpm), 30 °C, pH 5 (maintained by 27% ammonia solution). After the depletion of glycerol, two methanol additions (3 g/L final concentration) were performed to adapt the strain to methanol. After the second methanol depletion, the third addition of methanol was performed and a continual methanol feeding (methanol supplemented with 12 mL/L of PTM_1_) was started. The DO control was turned off, the agitation was set to 600 rpm, the temperature was lowered to 20 °C, and the pH was increased to 6. The automated program for methanol feeding was based on actual DO saturation according to Markošová et al. [38]. The fermentation was performed in duplicates for 164 h, after which one fermentation was terminated and the other continued for another 96 h. The cultivation medium was centrifuged (13,751× *g*, 10 °C, 15 min) and biomass was discarded.

The volumetric activity was defined as the amount of myrosinase in 1 mL of fermentation medium catalyzing the conversion of 1 μmol of sinigrin per minute. The specific productivity was defined as the amount of myrosinase produced in 1 L of fermentation medium per hour, which catalyzed the conversion of 1 μmol of sinigrin per minute.

### 3.3. Purification of Recombinant Myrosinase (myr-Δ19)

The supernatant containing myrosinase was pre-filtered using a wine filter and subjected to microfiltration on 0.2 μm membranes (Hydrosart^®^ Microfilter, Sartorius, Göttingen, Germany) using an ÄKTA flux (GE Healthcare, Chicago, IL, USA). The cell-free supernatant was desalted and concentrated 10-fold with a 30 kDa cut-off membrane (TANGEN XTM PRO PDn Cassette, ProStream, REPLIGEN, Waltham, MA, USA) with 50 mM sodium phosphate buffer, pH 6.5. A 5 mL aliquot of the desalted supernatant was diluted with MilliQ water to 30 mL and the pH was adjusted to 8 with 2 M NaOH. The supernatant was loaded onto a column (1.1 × 11.5 cm, flow 2.5 mL/min) of DEAE-Sepharose (Merck, Darmstadt, Germany) equilibrated with 25 mM Tris-HCl buffer, pH 8 (buffer A) [modified from Härtel and Brandt [22]]. Three concentrations of buffer B (250 mM NaCl in buffer A) were used to elute proteins from the column. Contaminating proteins were eluted from the column at 35% of buffer B (87.5 mM NaCl) for 8 CV, myrosinase was eluted at 60% of buffer B (150 mM NaCl) for 8 CV, and the most tightly bound proteins were eluted at 100% of buffer B. The myrosinase-containing fractions were concentrated and desalted with Amicon^®^ Ultra Centrifugal Filters, 30 kDa cut-off (Merck Darmstadt, Germany) and stored at −80 °C without glycerol.

### 3.4. Deglycosylation of Myrosinase (myr-Δ19)

Myrosinase was subjected to deglycosylation with EndoH (New England Biolabs, Ipswich, MA, USA) under denaturing conditions according to the manufacturer’s instructions. Under non-denaturing conditions, the reaction was performed at 30 °C for 16 h. After deglycosylation, myrosinase activity was measured and compared with a control sample, which was processed simultaneously without the addition of EndoH.

### 3.5. Myrosinase Activity Assay

All activity measurements in this paper were performed in a thermomixer (Thermomixer R, Eppendorf, Hamburg, Germany) with sinigrin as a substrate as described previously [13]. The kinetic parameters were determined at pH 7, 40 °C, and 1.5 mM of ascorbic acid with sinigrin concentrations of 0.25, 0.5, 0.75, 1, 1.5, and 3 mM. The sinigrin concentration was then analyzed by HPLC according to Tsao et al. [39]. One unit of myrosinase activity was defined as the amount of enzyme catalyzing the conversion of 1 μmol sinigrin per minute in 50 mM sodium phosphate buffer at pH 6.5 and 30 °C.

### 3.6. Analysis

Biomass growth was controlled by optical density (OD_600_) measurement at 600 nm (BioSpectrophotometer, Eppendorf, Hamburg, Germany). The concentration of glycerol and methanol in the cell-free supernatant during the fermentation was determined by HPLC (Agilent Technologies 1220 Infinity LC System with Agilent Technologies 1260 Infinity RI detector, Agilent Technologies, Santa Clara, CA, USA) using a WATREX Polymer IEX H form column and guard column. A flow rate of 0.8 mL/min of 9 mM sulfuric acid at 45 °C was used [38]. Sinigrin concentration was determined by HPLC (Agilent 1260 Infinity LC System with Quaternary Pump and UV detector, Agilent Technologies, Santa Clara, CA, USA) using a Phenomenex Gemini^®^ NX 5μm C18 110Å (150 × 4.6 mm) column according to Tsao et al. [39]. The nano-liquid chromatography and mass spectrometry analysis (LC-MS-MS) of the tryptic digest of myrosinase (*myr*-Δ19) was modified from [23]. The detailed method description can be found in Supplementary Materials. Protein concentration in cell-free supernatants was determined using the Bradford method [40]. Bradford reagent was purchased from Sigma-Aldrich (St. Louis, MO, USA).

## 4. Conclusions

In this study, an improved strategy for the extracellular production of recombinant myrosinase from *Arabidopsis thaliana* in *Pichia pastoris* KM71H is presented. The presence of a native N-terminal signal sequence in the myrosinase gene resulted in reduced production of the recombinant protein, even when the α-MF secretion signal was fused to the *At*TGG1 gene. The deletion of the native signal sequence significantly improved the secretion of recombinant myrosinase, resulting in a 40-fold increase in specific productivity. The purification of recombinant myrosinase was simplified after the signal sequence deletion as well. Using the presented production and purification strategy, large amounts of highly active and stable myrosinase can be produced, making myrosinase an interesting enzyme for applications in cancer therapy. This strategy can be applied for the recombinant production of other plant enzymes in *P. pastoris*.

## Figures and Tables

**Table 1:** The process parameters of the (α-MF)-driven extracellular expression of myrosinase in *Pichia pastoris* with (*myr*) and without (*myr*-Δ19) its native signal sequence in the expressed gene.

Gene	Maximal Specific Productivity ^1^ (U/L/h)	Activity ^1^ (U/mL)	Maximal Activity (U/mL)	Total Proteins ^1^ (mg/L)
*myr*	4.1 ± 0.2	0.67 ± 0.04	0.67 ^1^ ± 0.04	286.5 ± 21.8
*myr*-Δ19	164.8 ± 3.0	27 ± 0.4	37. 4 ^2^ ± 0.55	422.2 ± 18.8

^1^ detected in culture supernatant after 164 h of fermentation. ^2^ detected in culture supernatant after 236 h of fermentation.

## Data Availability

The data presented in this study are available on request from the corresponding author.

## Supplementary Materials

The following are available online at www.mdpi.com/1422-0067/22/21/11889/s1.
