# Supplementary material for: Improved Production of Recombinant Myrosinase in Pichia pastoris
Source: Int J Mol Sci. 2021 Nov 2;22(21):11889. doi: 10.3390/ijms222111889 (PMC8585081; doi:10.3390/ijms222111889)
Supplement: Supplementary file 1 [file ijms-22-11889-s001.zip › ijms-1400915-supplementary.pdf]

Supplementary information

S1: Cultivation media

S1.1 Preservation of *P. pastoris* KM71H (Mut^S):

P. pastoris was cryopreserved in YPD [Yeast extract/Peptone/Dextrose medium; 1% (w/v) yeast extract, 2% (w/v) peptone, and 2% (w/v) glucose] in 30% (v/v) glycerol; or kept on a Petri dish containing 2% (w/v) agar and 50 mg/L Zeocin™ (InvivoGen, USA).

S1.2 Cultivations in 96-deep well plates

BMD [Buffered Minimal Dextrose medium; 1.34% (w/v) YNB (Yeast Nitrogen Base), $4x10^{-5}$% (w/v) biotin, 1% (w/v) glucose, and 200 mM potassium phosphate buffer, (pH 6)],

BMM2 [Buffered Minimal Methanol medium; 1.34% (w/v) YNB, 4.10^{-5}% (w/v) biotin, 1% (v/v) methanol, 200 mM potassium phosphate buffer (pH 6)]

BMM10 [Buffered Minimal Methanol medium; 1.34% (w/v) YNB, 4.10^{-5}% (w/v) biotin, 5% (v/v) methanol, and 200 mM potassium phosphate buffer (pH 6)]

S1.3 Flask experiments

BMGY [Buffered Glycerol-complex Medium; 1% (w/v) yeast extract, 2% (w/v) peptone, 1.34% (w/v) YNB, 4×10^{-5}% (w/v) biotin, 1% (v/v) glycerol, and 100 mM potassium phosphate (pH 6)]

BMMH [Buffered Minimal Methanol Medium; 1.34% (w/v) YNB, 4×10^{-5}% (w/v) biotin, 0.5% (v/v) methanol, and 100 mM potassium phosphate (pH 6)]

S1.4 Fermentation experiments

BSM medium (Basal Salt Medium; per 1 L: 0.93 g $CaSO_4$, 18.2 g K_2SO_4, 14.9 g $MgSO_4$. 7 H_2O, 4.13 g KOH, 40 g glycerol, and 26.7 ml 85% H_3PO_4)

PTM1 (trace salts solution; per 1 L: 6 g $CuSO_4$ .5 H_2O, 0.08 g NaI, 3 g $MnSO_4.H_2O$, 0.2 g Na_2MoO_4.2 H_2O, 0.02 g H_3BO_3, 0.5 g $CoCl_2$, 20 g $ZnCl_2$, 65 g $FeSO_4.7H_2O$, 0.2 g biotin, and 9.2 g H_2SO_4) per litre of BSM medium.

S2: Nano-Liquid Chromatography and Mass Spectrometry Analysis (LC-MS-MS) of purified myrosinase (myr-Δ19)

The sample containing 250 μg of protein was digested using trypsin (Sigma-Aldrich, St. Louis, MO, USA) at a ratio of 1:25 (0.2 μg/μL trypsin) overnight at 37 °C. For protein analysis, a nano system liquid chromatograph (Ultimate 3000 RSLC, Thermo Fisher Scientific, Germering, Germany) and mass spectrometer with electrospray ionization (ESI) and a 3D ion trap mass analyzer (Amazon SL, Bruker, Bremen, Germany) were used. The mobile phase consisted of component A [0.1% formic acid (FA) in 2% acetonitrile (ACN)] and component B (0.1% FA in 95% ACN). The loading phase consisted of 0.05% FA in 2% ACN. The mobile phase flow rate was 0.4 μL/min with a gradient in length: 0–50% B for 90 min followed by 10 min at 95% B and 20 min at 4% B. The samples (2 μl) were injected in partial inject mode. The analyzed samples were separated on a 75 μm × 300 mm column with a 3 μm particle size at 100 Å and 40 °C. The spectrometer parameters were set as follows: 1450 V capillary voltage, 0.5 Ψ atomizer pressure, 3.0 L/min gas flow rate, 150 °C gas temperature, positive polarity, and scan range 50–2200 m/z at a scan rate of 8100 m/z/sec.

For database searching and protein identification the following programs were used: the Hystar program to define input LC-MS-MS method, the Data Analysis software to obtain spectra and chromatograms, the Proteinscape software for correlation analysis and the Mascot server 2.5.0. as a search library for protein identification. The parameters used in the Mascot search were the same as previously reported (Andelova et al., 2020).
